# Neonatal Assessment Manual Score: Is There a Role of a Novel, Structured Touch-Based Evaluation in Neonatal Intensive Care Unit?

**DOI:** 10.3389/fped.2020.00432

**Published:** 2020-08-06

**Authors:** Andrea Manzotti, Francesco Cerritelli, Marco Chiera, Erica Lombardi, Simona La Rocca, Pamela Biasi, Matteo Galli, Jorge Esteves, Gianluca Lista

**Affiliations:** ^1^RAISE Laboratory, Foundation COME Collaboration, Pescara, Italy; ^2^Division of Neonatology, “V. Buzzi” Children's Hospital, ASST-FBF-Sacco, Milan, Italy; ^3^Research Department, SOMA, Istituto Osteopatia Milano, Milan, Italy; ^4^Gulf National Centre, Foundation COME Collaboration, Riyadh, Saudi Arabia; ^5^Research Department, University College of Osteopathy, London, United Kingdom

**Keywords:** neonatology, manual assessment, haptic perception, body volume, autonomic nervous system, prematurity, touch, neonatal intensive care unit

## Abstract

Despite the technological improvements in monitoring preterm infants in the neonatal intensive care unit, routine care in the neonatal ward is primarily based on manual procedures. Although manual clinical procedures play a critical role in neonatology, little attention has been paid to palpation as a clinical assessment tool. Palpation is a clinical evaluation tool that relies mostly on the senses of touch and proprioception. Based on recent studies investigating the role and clinical effectiveness of touch in full-term and preterm babies, this paper proposes an evaluative touch-based procedure—the Neonatal Assessment Manual Score (NAME) model—that could be useful in the neonatal ward and describes its rationale. The operator applies gentle light pressures to the infant's body. In essence, the touch stimulates low-threshold afferent fibers that could influence the interoceptive cerebral network and the autonomic nervous system, thus altering the blood flow and breathing rhythm. These events could change how bodily fluids distribute among body segments and hence the body volume. The volume modification could be felt manually through haptic perception owing to the high sensitivity of the fingers. On the basis of their clinical conditions and stage of development, infants will respond differently to the applied pressures. Evaluating the infant's response, the operator produces a score of “bad,” “marginal,” or “good” for communicating quickly and clearly the infant's conditions to other professionals. Because the NAME model is intended for every professional who is used to touch-based procedures, if future studies confirmed its validity and reliability in clinical practice, the NAME model could become a part of the neonatal ward routine care for better assessing and managing the infant's conditions, even during emergencies.

## Introduction

In the last decades, newborns have been increasingly admitted to the neonatal intensive care unit (NICU) (1, 2). Different analyses showed that more than half of the infants admitted are full term and with appropriate birth weight (1, 2). Indeed, even full-term newborns can be negatively affected by maternal stress and labor complications (3, 4). In the first days of life, babies can suffer from several pathologies (e.g., early-onset sepsis, respiratory failure, and hypoxic–ischemic encephalopathy) or experience complications that require professional evaluation to prevent adverse outcomes (2). Among the difficulties that force a newborn to be admitted to a NICU, low birth weight and, in particular, preterm birth play central roles (1, 2).

Prematurity represents a global burden with one in 10 babies born preterm and one to three in 100 babies born before 32 weeks of gestation worldwide (5, 6). Prematurity entails both several short-term adverse outcomes (e.g., respiratory distress syndrome, sepsis, necrotizing enterocolitis, cardiovascular impairment, intraventricular hemorrhage, and periventricular leukomalacia) and long-term adverse outcomes (e.g., bronchopulmonary dysplasia, neurodevelopmental delay, reduced growth, and hearing and visual impairments) (6). Neonatologists need to carefully monitor the psychophysical development of preterm infants to reduce the risk of these consequences and to create therapeutic plans that respect the physiological growth and aim to improve the health of the newborns (7, 8).

Despite the technological advances made in the last decade to monitor preterm infants in the NICU, routine care is primarily based on manual procedures like changing diapers or performing heel sticks. It was calculated that, per day of hospitalization, nurses and physicians physically handle newborns more than a 100 times (9). However, touching has a specific aim—to perform health-care procedures like feeding, weighing, applying nasogastric tubes, changing diapers, performing venipunctures, and palliative care procedures, as well as handling emergencies (e.g., using tracheal tubes or nasal prongs for respiratory failure). Every procedure is acquired through specific training aimed at improving the technical execution (10), but it often overlooks the use of touch. Interestingly, in this context, touch is somehow non-specific, meaning that little or no attention is paid to how to touch and how touch can affect newborns. How NICU professionals touch newborns has been traditionally considered by many as irrelevant (11).

Different research groups started to investigate the role of touch and how touch is processed by full-term and preterm babies. Early “touch-based” studies used to explore the effect of touch associated with procedures. For example, changing diaper, a typical routine-care procedure, can induce pain-like behaviors in preterm infants, as per increased reflex behavior (e.g., withdrawal) after tactile stimulations (12, 13). The same touch can induce as much stress as invasive procedures (e.g., heel sticks and venipuncture) and make subsequent invasive procedures more painful for preterm infants, especially if unstable (13, 14). Indeed, the development and the conditions of the nervous system (from states of peripheral and central sensitization to specific pathologies) can greatly influence how to touch, and other stimuli can be perceived by newborns and infants (13, 14).

However, a closer look at the type of touch used showed that these research studies applied clustered care—grouping together several routine procedures including changing the diaper, measuring the abdominal girth, taking the body temperature, and cleaning the newborn's mouth or nose (13, 14). Even though used to reduce stressful episodes during the day, clustered care increased the expression of movements such as an extension of the arms and legs, finger splays, airplane (infant extends arms laterally), sitting on air, and salute (extension of the arms into midair in front of the infant); according to the Newborn Individualized Developmental Care and Assessment Program (NIDCAP®) model, which was primarily developed for taking care of preterm infants, these movements are important stress cues (13, 14).

Because other studies showed positive effects of clustered care in stable preterm infants, this conflicting evidence could be due to the lack of attention to how to properly handle preterm babies (13, 14). However, it is not easy to precisely disentangle the effect of touch from the other procedures.

Recent studies demonstrated that a more structured, patterned touch could induce positive effects on the newborns' health conditions. Gentle stroking can attenuate noxious-evoked brain activity during painful procedures, lower heart rate (HR), increase oxygen saturation (SpO_2_) as well as respiratory sinus arrhythmia (an index of vagal tone), and decrease crying time (15–18). A randomized controlled trial found that in the first 4 min after birth, 10 s of gently rubbing the back or the soles of the feet, alternated with 10 s of rest, significantly increased oxygenation in preterm infants between 27 and 32 weeks of gestational age, enhancing the spontaneous breathing and so the minute ventilation (19).

In synthesis, gently touching the babies induces a positive and relaxed state. Therefore, several kinds of “positive touch” have been proposed, such as gentle human touch: placing one hand on the head and the other hand on the abdomen (16); parental touch and caresses (17); dynamic or affective touch (18); osteopathic manipulative treatment (20); and kangaroo care (21) as well as facilitated tucking, rocking, holding, swaddling, and massage-like touch (11). All these types of touch consist of slow and gentle strokings or pressures, aiming to promote preterm infants' health and growth.

Although the evidence on touch-based procedures in neonatology, especially in the NICU scenario, is still scarce, some initial evidence is emerging on clinical outcomes associated with positive touch. However, all studies focus their attention on the use of touch as a “treatment” procedure. There is a lack of research on the use of touch as an assessment tool to obtain the newborn's necessary clinical information, which might lead to improving the newborn's daily care plans. NICU staff can use several assessment methods to get information about the infant's development, growth, and neurobehavioral functioning. Some among the most valid and reliable assessment procedures are the modified Pain Assessment Tool (22), the Test of Infant Motor Performance (23), the Alberta Infant Motor Scale (24), the Neonatal Behavioral Assessment Scale (25), and the Assessment of Preterm Infants' Behavior (26). A closer look at these methods showed that touch is sometimes used, but only for evaluating muscle tone in case of pain or specific behaviors (e.g., newborns' ability to motor control or to console themselves). Besides, these tools are primarily based on visual observations, whose objectivity could be improved through a more direct quantification method.

Despite the number of assessing methods used in neonatology, none suggested a structured touch-based evaluation. To this end, the present paper aims to fill this gap by proposing a manual assessment for evaluating the infants also during the NICU hospitalization and discussing its rationale.

## The Neonatal Assessment Manual Score Model

### Overview of the Neonatal Assessment Manual Score Model

The Neonatal Assessment Manual Score (NAME) model was developed to manually evaluate infants, including preterm ones, within the NICU setting. This clinical evaluation tool is aimed at every manual therapist (i.e., osteopaths, physiotherapists, or chiropractors) and NICU professional (i.e., nurses and physicians) who use touch-based procedures during their routine care.

**Figure 1:**
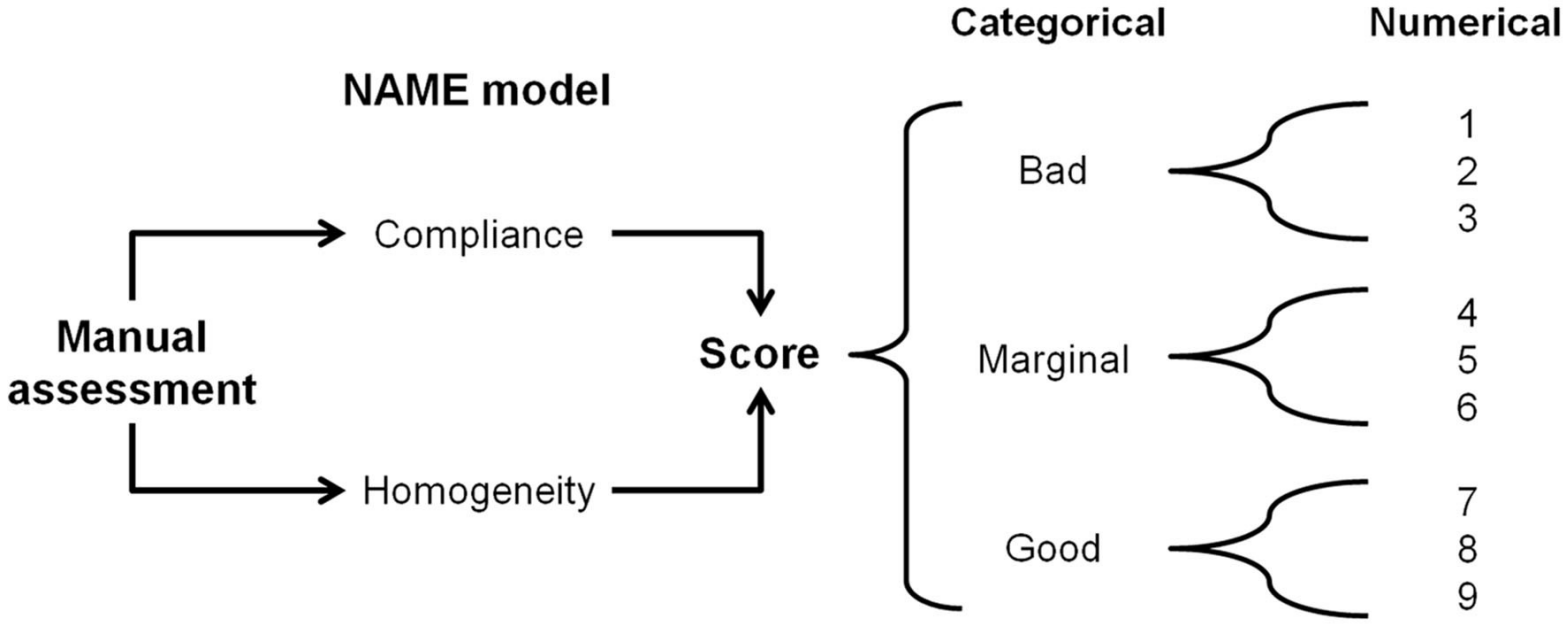
The NAME model. Through a manual palpatory assessment, the operator evaluates how the infant's body responds to mechanical stimuli and gives a categorical score to define the response. The categorical NAME is the main score to be used for communication inside the NICU. The same operator then converts the categorical score into a numerical one, which aims to better monitor the infant's conditions in the long term. NAME, Neonatal Assessment Manual Score; NICU, neonatal intensive care unit.

The NAME model is a touch-based manual palpation examination tool that produces two scores/indexes: (1) categorical, with three levels (“bad,” “marginal,” and “good”) and (2) numerical, a 1-to-9 Likert scale, where 1–3 correspond to bad, 4–6 marginal, and 7–9 good (see Figure 1). The categorical score is the NAME model's primary score—it is a rapid and accurate vehicle to communicate the infant's conditions among different professionals. The categorical rating is particularly useful during the handling of emergencies when speedy and efficient communication is paramount. The numerical score enables professionals to effectively monitor the evolution in the infant's conditions during hospitalization by better defining the infant's health condition for every NAME category in three numerical sublevels.

The NAME manually evaluates how the infant responds to an external stressor such as static touch. In particular, the operator evaluates how the infant reacts to light mechanical sensory stimuli applied to different bodily areas. The operator assesses two parameters: (a) compliance, that is, whether the body changes its volume accordingly to the applied mechanical stimuli (pressure and distension); and (b) homogeneity, that is, whether the infant's body tissues adapt to the mechanical stimuli in the same way throughout their body—see the following sections for a further detailed explanation. The estimated time to perform the NAME is about 90 s.

### Description of the Neonatal Assessment Manual Score

The NAME procedure is used within the NICU where babies are approached while they are in the incubators or open beds. The assessment can be performed anytime during the day when the baby is in a quiet and calm state; because it takes a small amount of time, it can be performed routinely with every physical examination.

**Figure 2:**
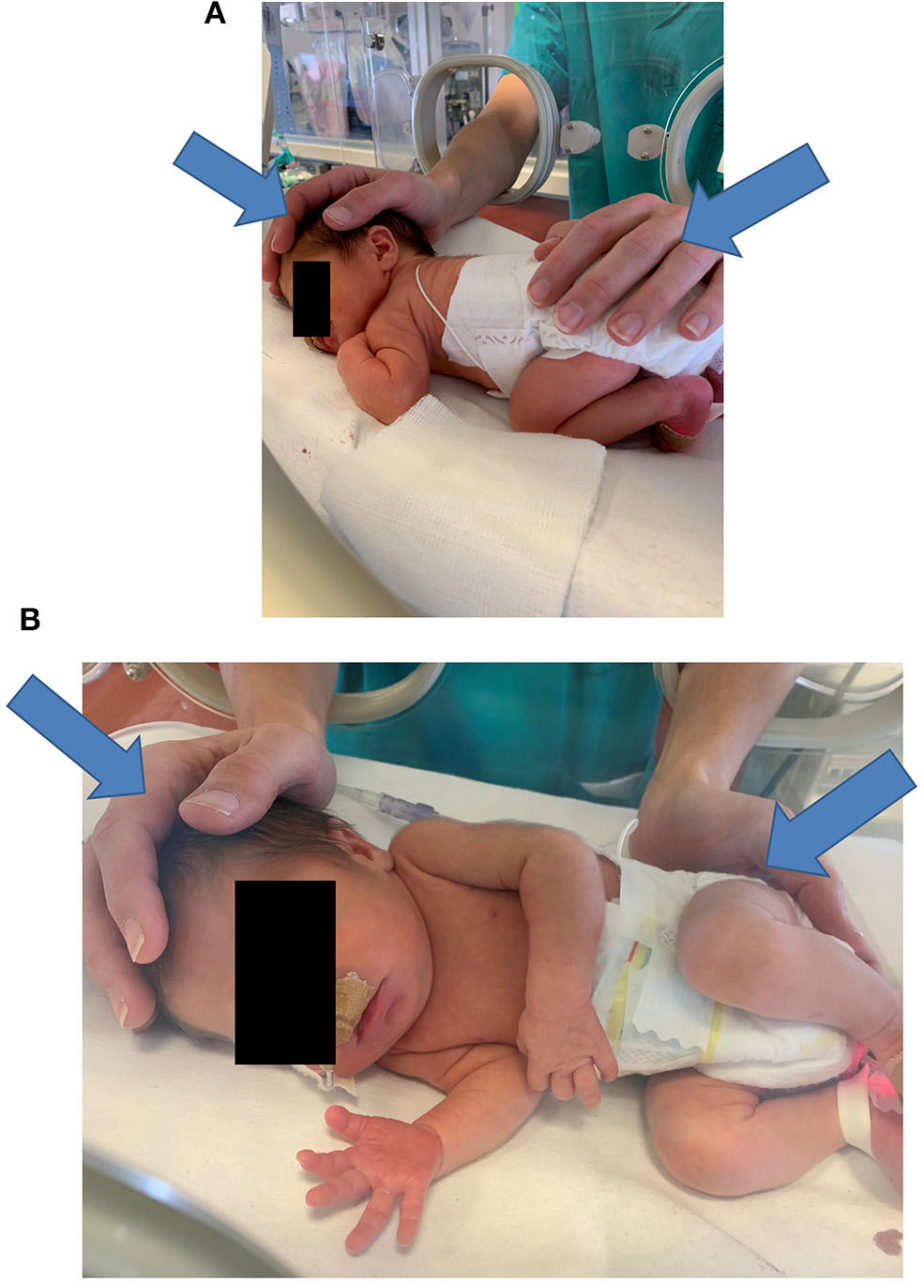
Typical hand positioning for a NAME procedure **(A,B)**. In **(A,B)** the arrows indicate the direction of the manual stimuli. NAME, Neonatal Assessment Manual Score.

The operator places both hands on the infant—one hand touches, that is, palpates the cranial region with the whole palm, while the other hand touches the sacral crest with the proximal phalanges (see Figure 2). If this approach is not feasible, the operator places two hands on opposite body areas to have the infant's body between them. The operator induces precise mechanical stimuli as detailed below and then focuses on interpreting the sensory signals received primarily via their haptic system, that is, touch and proprioception. This interpretation is focused mainly on how the infant's body reacts to the mechanical stimuli, in particular, how the tissues change their softness.

The NAME procedure consists of two phases.

The first evaluation phase lasts ~10 s and assesses the infant's general compliance. The operator applies a light pressure with both hands and then releases it. The operator focuses on perceiving the resistance, determined as softness or hardness, that is, how the infant's body as a whole responds to the manual pressure.

The second evaluation phase lasts ~80 s and assesses the infant's homogeneity, that is, whether the baby displays the same softness throughout their body. Using the palm and the phalanges of both hands, the operator applies the same light pressure as before but focuses instead on interpreting changes in the softness of the tissues across the infant's body. During this phase, the operator can identify body areas that react differently among them, that is, regions that display different tissue softness. These areas can define regions of interest (ROIs) that could correlate with infant's clinical conditions.

After the two evaluation phases, the operator gives a score to define the infant's responses to the test (see Figure 1).

The operator needs specific training to understand the correct manual procedure to perform. A similar pre-training was done in two previous studies in which professionals with more than 5 years' experience in the NICU used the NAME procedure as the evaluation phase (18, 27).

### Rationale for the Development of the Neonatal Assessment Manual Score Model

As a clinical palpation evaluation tool, the NAME model uses touch and manual pressure as many other routine care procedures. However, there is a lack of debate in the literature regarding the type of touch to be used and how light touch may induce changes in the infant's body tissues. Moreover, there is a need to critically discuss whether and how the operator may manually feel these physiological changes.

#### Afferent Systems and Central Elaboration

The nervous system can detect tactile stimuli through several skin mechanoreceptors that convey the stimuli via the spinal cord to the brain. Whereas, sensory discrimination in the brain is conveyed by the discriminative/sensory or A system, the evaluation of affective/emotional qualities is conveyed by the affective/homeostatic or B system (28, 29).

The A system (laminae III–V of the spinal cord, thalamus, and the primary somatosensory cortex) recognizes the sensory features of mechanical stimuli. It relies on rapid and heavily myelinated Aβ afferent fibers, which innervate several low-threshold mechanoreceptors (LTMs). Among the LTMs, the Merkel discs detect stimuli that are perceived as static pressure (28, 29).

The B system (laminae I and II of the spinal cord and cerebral areas, which span from the brainstem to the insular cortex) represents the primary substrate for interoception—the process used by the brain to detect every physicochemical stimulus in the body (e.g., mechanical stress, temperature, and oxygen concentration) to regulate the organism arousal, autonomic functions, and hypothalamus–pituitary–adrenal axis, hence the name interoceptive or “homeostatic system.” The cerebral areas that control the B system are also involved in the conscious perception of emotions and social interactions, hence the name “affective” system [(29); for further details about interoception, autonomic nervous system (ANS), and touch, see (30)]. The B system relies on slow and thin low-myelinated Aδ and unmyelinated C afferent fibers that can be high- or low-threshold receptors (28). Especially in hairy skin, a group of C fibers named C-tactile (CT) are particularly sensitive to low-threshold mechanical stimuli: slow (1–10 cm/s) and light (0.2 mN–2.5 N) strokings that give the perception of pleasure. This kind of touch resembles the parents' caresses on their babies (17, 28, 31). CT fibers also seem to respond to light static pressure (32, 33).

Therefore, gentle and static touch can recruit both the Merkel discs (discriminatory system) and CT fibers (affective system).

#### Changes in Body Tissues

From a physiological standpoint, owing to their specific characteristics, Merkel's discs and CT fibers can induce local and central interoceptive-based responses.

Through the Merkel–neurite complex (MNC), Merkel's disc stimulation can release vesicles containing glutamate, serotonin, substance P, vasoactive intestinal peptide, and calcitonin gene-related peptide (34)—substances that can influence tissue blood circulation, local and pulmonary hemodynamics, and smooth musculature activity (35–37). Interestingly, Merkel's discs are increasingly recognized to have neuroendocrine activity (38): some of the neuropeptides they release can indeed trigger the B system (28). Thus, the Merkel receptors might be considered the connecting point between the A and B systems.

CT fibers can influence the interoceptive network and the ANS activation (30, 32), thus inducing local and central changes in the infant's blood flow, blood pressure, HR, breathing pattern, and SpO_2_ (18, 31, 39).

When blood flow and breathing pattern change, body volume—how bodily fluids distribute in the various body segments and tissues—changes accordingly. Body volume and composition can be measured to assess the infant's health, development, and growth (40), thus giving useful information as the monitoring of neonatal hemodynamics (e.g., systemic and pulmonary blood flow and vascular resistance) (41, 42). Hemodynamics is indeed continuously monitored because abnormal cardiovascular function during the neonatal period correlates with a higher risk of morbidity and mortality (41, 42).

The ANS functioning plays a central role in directing these changes: indeed, different infants can show even opposite hemodynamic modifications, which are entirely different changes in body volume, due to the individual ANS state of development (41, 42).

During the third trimester of gestation and the perinatal period, the ANS undergoes significant modifications: both the sympathetic and parasympathetic activities rise. At birth, the ANS shows a sympathetic predominance, whereas after birth, the parasympathetic branch starts to take over (39, 43). The nervous system development correlates with the increase in fetal body movements, especially thoracic–respiratory ones, and the appearance of both respiratory sinus arrhythmia and baroreflex mechanisms (44). These motor and cardiorespiratory changes are considered the basis for the future fight-or-flight response (45), and the ANS development correlates with an increase in HR variability, an index of the adaptive regulation processes efficacy (39). In the perinatal period, the ANS development and the infant's adaptation capacity, especially cardiorespiratory, are thus strictly tied between them.

The ANS development is, however, delicate, and it can be disrupted by several events, such as maternal stress, nutritional deficiency, premature birth, complicated labor, immediate or delayed cord clamping, and NICU invasive procedures (39, 43). A disrupted ANS reduces the newborn's capacity to efficiently adapt to its environment, especially when it is continuously changing and challenging as in NICU (39). A disrupted ANS means thus a disrupted cardiorespiratory adaptation.

Therefore, due to the ANS involvement in light touch, the same gentle manual pressure could induce different changes in hemodynamics (e.g., HR, HR variability, SpO2, and pulmonary and systemic blood flow) and then in body volume, in different infants with different nervous development and clinical conditions.

#### Haptic Perception

When an operator touches a newborn, he or she induces an ANS activation. Reaching a judgment of changes in the newborn's body tissues requires an ongoing interaction between ascending (i.e., bottom-up processing) and descending mechanisms (i.e., top-down processing) in the operator's nervous system (46). What we perceive is a fine balance between top-down knowledge-based prediction and bottom-up incoming sensory evidence (47). In this particular context, sensation refers to the detection of a change in the newborn's body. In contrast, perception relates to the interpretation of that change in terms of compliance and homogeneity. Judgments of changes in the newborn's body are therefore likely to influenced by predictions made about the upcoming bottom-up sensory signals [see (48), on this point].

Bottom-up processing begins with the activity of sensory receptors located in the operator's hands and using the haptic system. The haptic system is a perceptual system mediated by two afferent subsystems, cutaneous and kinesthetic, that typically involve active manual exploration (49). The haptic system has perceptual and memory functions involved in recognition of object shape and microgeometric tissue properties. Haptic perception constitutes the foundation of manual clinical evaluation, in particular for the assessment of tissue compliance—how the tissues respond to palpation characterized by light pressure (50). Haptic perception is a delicate process: the human fingers—in particular, the index finger—can touch and discriminate surfaces with small wrinkles of various amplitudes (51). The fingertips also display low thresholds for discriminating objects with different volumes (52).

Incoming sensory signals conveyed via the haptic system are processed in cortical areas responsible for sensory discrimination, object recognition, and decision making (46). The interaction between top-down predictions and bottom-up incoming sensory information is critical to the operator's decision making regarding changes in volume and tissue compliance.

Volume perception of an object is essential to grasp its spatial properties, and it is influenced by several material properties of the touched object, in particular by compliance. Compliance is the object's ability to deform. It determines the softness of an object: it also depends on the amount of contact area between the object and the hand, and the distance that a fingertip penetrates an object (53–55). The object compliance can also be felt at a distance—without direct contact—if something lays between the hand and the object, for example, a stylus used for reaching a distant object (55, 56). For the infant's body, the body itself can be the “stylus” to feel the compliance of internal tissues and how the applied pressure spreads throughout the tissues. In essence, the body constitutes a tensegrity structure in which mechanical stress distributes throughout the whole structure of bones, muscles, and connective tissues (57).

The sensitivity of the hand LTMs and the specialization of the haptic system together with the accumulated professional knowledge in the NICU setting will contribute to effective interaction between top-down predictions and bottom-incoming sensory signals processed and modulated at different levels of the nervous system hierarchy. It is therefore plausible that an operator may feel the changes in the newborn's body volume, evaluating the compliance of the newborn's tissues, that is, perceiving the resistance put up to the applied pressures and the changes in tissue softness (51, 52, 55).

#### The Force Needed for Neonatal Assessment Manual Score Model

What is the exact amount of force that a hand has to apply to engage the infants' deep tissues? What is the force needed to induce changes in the infants' deep tissues that are manually perceivable? Even though light forces can stimulate Merkel's discs and CT fibers, it is dubious that these forces can engage deeper body tissues and CT fibers. To the best of our knowledge, no studies have investigated this issue. However, just a little higher force may be sufficient. Studies on adult cadavers showed that a force of 10 N applied to the back can transmit to a point 8–10 cm far away (58, 59). According to a 3D mathematical model of fascial connective tissue, a pressure of 4–5 N/cm^2^ can deform the superficial nasal fascia. In essence, for the general fascia, the pressure needed is higher, but the NAME aims to engage the tissues, not to modify them (60).

Therefore, to better grasp the amount of force needed for the NAME model, we performed some empirical studies with healthy newborns—we found that a force of 10 ± 2 N applied with a hand is sufficient to manually feel, through haptic perception, changes in the softness of both superficial and deep body tissues. Hence, we can also hypothesize that a force of 10 N can stimulate both superficial and deep CT fibers.

#### Summary of the Neonatal Assessment Manual Score Conceptual Framework

**Figure 3:**
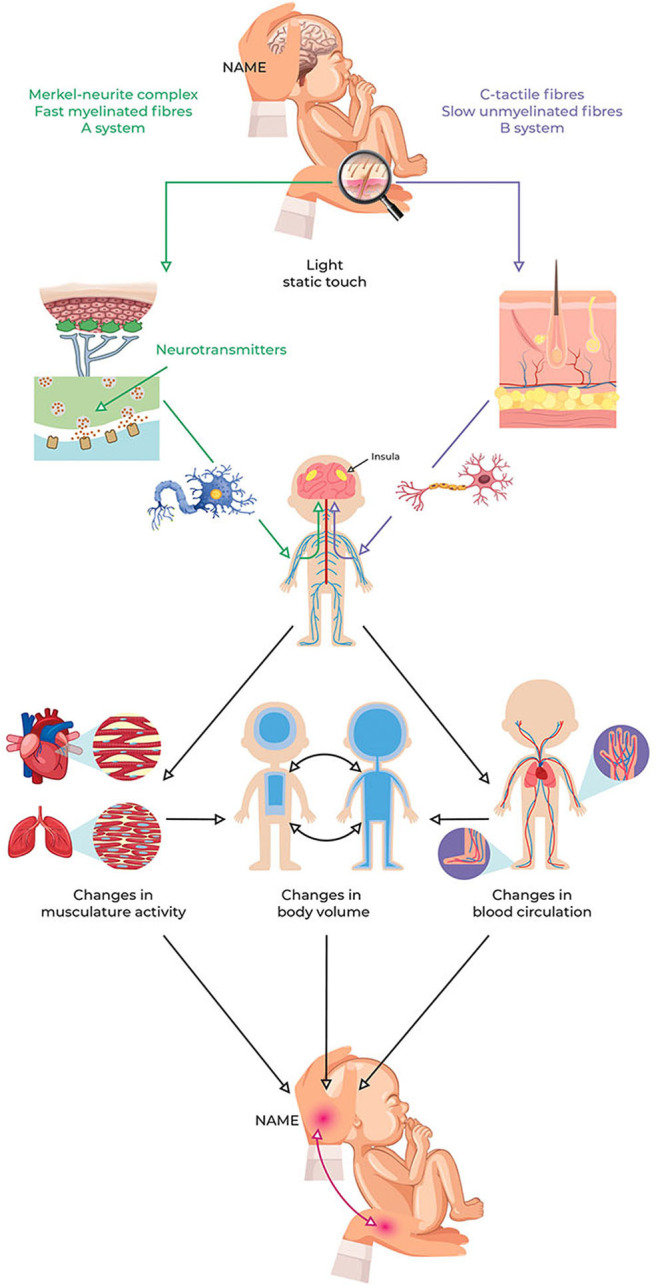
NAME model conceptual framework. Light pressure can stimulate peripheral LTMs and then induce local and central changes due to ANS reflexes and interoception involvement. These changes influence the cardiovascular and respiratory systems, thus modifying the redistribution of body fluids. The redistribution of body fluids represents a change in the body volume that can be perceived through the haptic system as softness. The NAME comes from evaluating if the body complies with manual pressure, both globally and locally. Because the redistribution of body fluids is usually measured to assess the infant's clinical conditions, perception via the haptic system could also help to recognize them. ANS, autonomic nervous system; NAME, Neonatal Assessment Manual Score; LTMs, low-threshold mechanoreceptors.

The NAME procedure can be summarized based on the following conceptual framework (see Figure 3):

- A little amount of pressure applied on the infant's body can stimulate the Merkel discs and CT fibers.
- Merkel's discs and CT fibers can elicit ANS responses that activate local and central interoceptive-based responses, which affect the cardiovascular and respiratory systems (e.g., change in HR, blood flow, and breathing pattern) within seconds.
- Based on the infant's ANS development and maturation, cardio-respiratory and hemodynamics changes bring about different body volume changes.
- The body volume changes can be perceived through the haptic system due to the fingers and fingertips high sensibility and involving interactions between top-down predictions and bottom-up sensory signals.
- Through changes in tissues softness, a professional can recognize compliance (if the infant's whole body adapts accordingly to the manual stimuli) and homogeneity (if the infant's tissues adjust to the mechanical stressors in the same way throughout the body) of the infant's responses.
- For compliance, the operator evaluates the resistance (assessed as softness or hardness) the infant's body as a whole put up to manual pressure, whereas for homogeneity, the operator compares the softness changes felt by the palms and fingertips across the infant's body.
- Because body volume is often measured to assess the infant's health and growth, it can be argued that the perceived changes in body volume reveal the newborn's general condition. In essence, it might reflect, therefore, how the infant's nervous system elaborates the tactile stimuli and responds to them.

The purpose of the NAME model is to enable professionals to perceive the infant's body compliance and homogeneity, which could reflect how the infant adapts to external stressors and the infant's ANS functioning and general health condition. Because ANS and body volume changes reflect infants' clinical conditions, babies with a good development will show a tissue response both compliant and homogenous (they adapt to the stimuli through harmonic body responses), whereas babies with developmental problems will not show a tissue response either compliant or homogenous (they fail to adapt to the sensory stimuli).

## Discussion

### Significance and Meaning of the Neonatal Assessment Manual Score Model

The present paper presents the rationale and underpinning theory behind the NAME model; a new suggested manual palpatory assessment to be used in the neonatal ward. NICU professionals use their hands to touch infants hundreds of times per day (9) and to perform technical procedures such as venipuncture and heel sticks (10). Notwithstanding this, they lack awareness about how touch can affect infants and how they can use touch to assess infants.

Touch can induce changes in the infant's body, and the NAME aims to show that these changes can be perceived primarily through the haptic system and be correlated with the infant's clinical conditions. In particular, the NAME model aims to assess two critical adaptive processes: a global adaptation (whether the body follows the manual stimuli given or puts up some resistance—compliance evaluation) and a local adaptation (whether different body areas display the same softness—homogeneity evaluation). Healthy infants will show high compliance and the same softness to the mechanical stimuli throughout their whole body. In contrast, clinically complicated infants will fail to be compliant or show body areas with different levels of softness/stiffness.

In particular, owing to incomplete ANS development, we expect very early preterm newborns to show low compliance and homogeneity more likely than full-term newborns. The latter should have good probabilities of showing high compliance and homogeneity. Between these extremes, we expect newborns to respond differently based on their nervous development. Besides, at a given age, newborns can also respond differently to the manual stimuli owing to differences in clinical conditions.

We also expect that the present perspective paper can clarify what happens in infants when they are touched—for several years even the potentially noxious effects of painful procedures were under-considered in the care of infants (11).

### Limitations

The NAME relies on judgments regarding changes in tissues texture and softness by using the haptic system primarily. In analogy to other sensory systems, the information conveyed by the haptic system is subject to several top-down processes and biases. For example, the perception can be modified by expectations of tissue softness according to past experiences (61), visual signals, or the movement of another part of the operator's body (62). Hence, the haptic perception has to be refined to exclude every possible distraction: if every operator can feel subtle volume changes, training will help to interpret them better (50). Experience in the neonatology ward and touch-based procedures could also help to reduce biases.

Concerning the NAME rationale, there are several gaps in the literature regarding the effects of touch in infants, particularly in preterms. It is, therefore, paramount to deepen what we have described in the present paper. For instance, the correlation between touch, ANS reactivity, and changes in body fluid distribution needs to be precisely determined, as well as the relationship between changes in body fluid distribution and infant's clinical conditions.

Beyond defining a rationale, we must ascertain the NAME validity and reliability. In essence, an evaluative procedure needs to measure what it proposes to measure, and it needs to reproduce consistent and coherent results at different times and among different operators (63).

Moreover, it is not clear if the NAME could be useful to the NICU staff to perceive what happens in neonates when they are touched in the course of pharmacological sedative or analgesic management, in terms of efficacy of analgesic drug therapy.

### Benefits

In contrast to widely used assessment procedures, such as the Alberta Infant Motor Scale (24) or the Assessment of Preterm Infants' Behavior (26), the NAME has the advantage of taking less time to be performed (see Table 1). Except for pain assessment (22), those procedures need time to be completed: from 30 min (for assessing infants) to several hours (for writing the clinical report) (23, 26). Besides, although haptic perception can be subjective to bias as detailed above, the training needed for performing the NAME model, the direct contact with the baby, and the fewer items to assess (i.e., compliance and homogeneity) could make the NAME model more objective than other scoring systems based on long visual observation.

**Table 1:** Briefly comparison between the NAME model and other assessment models used in the NICU (14, 22–26).

**Model**	**Time**	**Procedure (summary)**	**Purpose**
NAME	90 s	Stimulation through light manual pressure and haptic perception of bodily changes	To evaluate global adaptation and possibly predict clinical conditions
PAT	Time of the event evaluated	Visual observation on the infant's activity and assessment of vital signs	To assess pain and choose between nursing comfort measure or analgesia
TIMP	25–35 min	Visual observation of spontaneous infant's activity	To assess posture and selective control of movement
AIMS	20 min	Visual observation of spontaneous infant's activity	To evaluate gross motor maturation
NBAS	20–30 min	Multisensorial and social stimulation for evaluating infant's responses	To evaluate the infant's behavior and neurological status
APIB	45 min to 3 h	Multisensorial and social stimulation for evaluating infant's responses	To assess neurodevelopment and infant competence
NIDCAP®		Multisensorial stimulation and social for both evaluating infant's responses (such as APIB) and reducing stress	To assess the infant's behavior and provide supportive care for infants

*AIMS, Alberta Infant Motor Scale; APIB, the Assessment of Preterm Infants' Behavior; NAME, Neonatal Assessment Manual Score; NBAS, Neonatal Behavioral Assessment Scale; NIDCAP®, Newborn Individualized Developmental Care and Assessment Program; PAT, Pain Assessment Tool; TIMP, Test of Infant Motor Performance; NICU, neonatal intensive care unit*.

In comparison with a previous attempt (64), the NAME model is simpler because it involves static and gentle touch that can be performed by every professional that is already accustomed to touch-based procedures. Its simplicity can also make NAME a part of the routine procedure used in neonatology to assess the infant's conditions.

The homogeneity assessment could quickly identify ROIs in the infant's body. The clinical conditions of preterm infants can be very different in terms of nature and severity of symptoms; it would be interesting if these features could reflect how the body adapts to external mechanical stimuli and if every condition could reflect on a particular body area—an ROI—like referred pain, for instance. Because the body can be viewed as a tensegrity structure that always responds as a whole and in which every tissue is mechanically connected (57), this correlation could be possible. It constitutes a hypothesis we should test in the future and more clinical-oriented trials.

## Conclusions and Future Perspectives

The present paper aimed to define the rationale for a possible new manual assessment in the neonatology field—the NAME model. This procedure is intended for every NICU professional, and it aims to optimize the care of the infants in the neonatal ward.

Future studies will evaluate the validity and reliability of the NAME model in clinical practice to define its clinical usefulness. As every manual assessment, the NAME procedure is exposed to top-down biases, as discussed in the limitations section. However, because the operator needs to be experienced in touch-based procedures and the neonatology field, and to follow a specific training to understand the correct manual procedure to perform, and because this manual evaluation was used in two previous studies (18, 27), we expect the NAME to be reliable and valid.

Besides, future studies will test the predictive capacity of the NAME model, that is, whether the score correlates with or can predict the infant's clinical conditions, or at least his or her developmental trajectory. Because the NAME aims to evaluate changes in body volume that depend on the infant's ANS development and adaptive capacity, there is the chance that the score could inform about how the infant's conditions are evolving. If future evidence should support this hypothesis, then it would become interesting to compare the NAME model with other assessment procedures already used in the NICU with good predictive capacity. Such a comparison would better define the NAME clinical relevance and usefulness as a procedure that can assess the actual infant's conditions or also predict their development.

## Data Availability Statement

All datasets presented in this study are included in the article/supplementary material.

## Ethics Statement

Written informed consent was obtained from the individual(s), and minor(s)' legal guardian/next of kin, for the publication of any potentially identifiable images or data included in this article.

## Author Contributions

GL and JE revised and edited the final draft of the manuscript. All authors approved the final manuscript as submitted, agreed to be accountable for all aspects of the work, and contributed equally to the conceptualization and to the writing of the manuscript.

## Conflict of Interest

The authors declare that the research was conducted in the absence of any commercial or financial relationships that could be construed as a potential conflict of interest.

## Abbreviations

ANS: autonomic nervous system
CT: C-tactile
HR: heart rate
LTM: low-threshold mechanoreceptor
MNC: Merkel–neurite complex
NAME: Neonatal Assessment Manual Score
NICU: neonatal intensive care unit
ROI: region of interest
SpO_2_: partial oxygen saturation.

## References

[1] Harrison W Goodman D. Epidemiologic trends in neonatal intensive care, 2007-2012. JAMA Pediatr. (2015) 169:855–62. 10.1001/jamapediatrics.2015.1305 26214387

[2] Schulman J Braun D Lee HC Profit J Duenas G Bennett MV. Association between neonatal intensive care unit admission rates and illness acuity. JAMA Pediatr. (2018) 172:17–23. 10.1001/jamapediatrics.2017.3913 29181499 PMC5833518

[3] Babenko O Kovalchuk I Metz GA. Stress-induced perinatal and transgenerational epigenetic programming of brain development and mental health. Neurosci Biobehav Rev. (2015) 48:70–91. 10.1016/j.neubiorev.2014.11.013 25464029

[4] Tribe RM Taylor PD Kelly NM Rees D Sandall J Kennedy HP. Parturition and the perinatal period: can mode of delivery impact on the future health of the neonate? J Physiol. (2018) 596:5709–22. 10.1113/JP275429 29533463 PMC6265543

[5] Granese R Gitto E D'Angelo G Falsaperla R Corsello G Amadore D. Preterm birth: seven-year retrospective study in a single centre population. Ital J Pediatr. (2019) 45:45. 10.1186/s13052-019-0643-9 30971310 PMC6458791

[6] WHO World Health Statistics 2016. Monitoring Health for The SDGs. (2016). Available online at: http://apps.who.int/iris/bitstream/10665/206498/1/9789241565264_eng.pdf (accessed March 25, 2020).

[7] Fairchild KD Lake DE. Cross-correlation of heart rate and oxygen saturation in very low birthweight infants: association with apnea and adverse events. Am J Perinatol. (2018) 35:463–9. 10.1055/s-0037-1608709 29141263 PMC6543270

[8] Paviotti G de Cunto A Moressa V Bettiol C Demarini S. Thoracic fluid content by electric bioimpedance correlates with respiratory distress in newborns. J Perinatol. (2017) 37:1024–7. 10.1038/jp.2017.100 28749485

[9] Zahr LK Balian S. Responses of premature infants to routine nursing interventions and noise in the NICU. Nurs Res. (1995) 44:179–85. 10.1097/00006199-199505000-00009 7761295

[10] Marc-Aurele KL English NK. Primary palliative care in neonatal intensive care. Semin Perinatol. (2017) 41:133–9. 10.1053/j.semperi.2016.11.005 28162789

[11] Pillai Riddell RR Racine NM Gennis HG Turcotte K Uman LS Horton RE Non-pharmacological management of infant and young child procedural pain. Cochrane Database Syst Rev. (2015) 2015:CD006275 10.1002/14651858.CD006275.pub2 PMC6483553 26630545

[12] Fitzgerald M. The development of nociceptive circuits. Nat Rev Neurosci. (2005) 6:507–20. 10.1038/nrn1701 15995722

[13] Holsti L Grunau RE Whifield MF Oberlander TF Lindh V. Behavioral responses to pain are heightened after clustered care in preterm infants born between 30 and 32 weeks gestational age. Clin J Pain. (2006) 22:757–64. 10.1097/01.ajp.0000210921.10912.47 17057556 PMC1851898

[14] Holsti L Grunau RE Oberlander TF Whitfield MF Weinberg J. Body movements, an additional important factor in discriminating pain from stress in preterm infants. Clin J Pain. (2005) 21:491–8. 10.1097/01.ajp.0000146163.30776.44 16215334 PMC1852478

[15] Gursul D Goksan S Hartley C Mellado GS Moultrie F Hoskin A. Stroking modulates noxious-evoked brain activity in human infants. Curr Biol. (2018) 28:R1380–1. 10.1016/j.cub.2018.11.014 30562526 PMC6303187

[16] Herrington CJ Chiodo LM. Human touch effectively and safely reduces pain in the newborn intensive care unit. Pain Manag Nurs. (2014) 15:107–15. 10.1016/j.pmn.2012.06.007 24602430

[17] Van Puyvelde M Collette L Gorissen AS Pattyn N McGlone F. Infants autonomic cardio-respiratory responses to nurturing stroking touch delivered by the mother or the father. Front Physiol. (2019) 10:1117. 10.3389/fphys.2019.01117 31555148 PMC6724449

[18] Manzotti A Cerritelli F Esteves JE Lista G Lombardi E La Rocca S. Dynamic touch reduces physiological arousal in preterm infants: a role for C-tactile afferents?. Dev Cogn Neurosci. (2019) 39:100703. 10.1016/j.dcn.2019.100703 31487608 PMC6969366

[19] Dekker J Hooper SB Martherus T Cramer SJE van Geloven N Te Pas AB. Repetitive versus standard tactile stimulation of preterm infants at birth - a randomized controlled trial. Resuscitation. (2018) 127:37-43. 10.1016/j.resuscitation.2018.03.030 29580959

[20] Lanaro D Ruffini N Manzotti A Lista G. Osteopathic manipulative treatment showed reduction of length of stay and costs in preterm infants: a systematic review and meta-analysis. Medicine. (2017) 96:e6408. 10.1097/MD.0000000000006408 28328840 PMC5371477

[21] Boundy EO Dastjerdi R Spiegelman D Fawzi WW Missmer SA Lieberman E. Kangaroo mother care and neonatal outcomes: a meta-analysis. Rev Artic Pediatr. (2016) 137:e20152238. 10.1542/peds.2015-2238 26702029 PMC4702019

[22] O'Sullivan AT Rowley S Ellis S Faasse K Petrie KJ. The validity and clinical utility of the COVERS scale and pain assessment tool for assessing pain in neonates admitted to an intensive care unit. Clin J Pain. (2016) 32:51–7. 10.1097/AJP.0000000000000228 25756556

[23] Campbell SK Wright BD Linacre JM. Development of a functional movement scale for infants. J Appl Meas. (2002) 3:190–204. 12011500

[24] Jeng SF Yau KI Chen LC Hsiao SF. Alberta infant motor scale: reliability and validity when used on preterm infants in Taiwan. Phys Ther. (2000) 80:168–78. 10.1093/ptj/80.2.168 10654063

[25] Barbosa M Moreira J Tronick E Beeghly M Fuertes M. Neonatal behavioral assessment scale (NBAS): confirmatory factor analysis of the six behavioral clusters. Early Hum Dev. (2018) 124:1–6. 10.1016/j.earlhumdev.2018.07.007 30075392

[26] Als H Butler S Kosta S McAnulty G. The assessment of preterm infants' behavior (apib): furthering the understanding and measurement of neurodevelopmental competence in preterm and full-term infants. Ment Retard Dev Disabil Res Rev. (2005) 11:94–102. 10.1002/mrdd.20053 15856436 PMC4106135

[27] Manzotti A Cerritelli F Lombardi E La Rocca S Chiera M Galli M. Effects of osteopathic treatment versus static touch on heart rate and oxygen saturation in premature babies: a randomized controlled trial. Complement Ther Clin Pract. (2020) 39:101116. 10.1016/j.ctcp.2020.101116 32379655

[28] McGlone F Wessberg J Olausson H. Discriminative and affective touch: sensing and feeling. Neuron. (2014) 82:737–55. 10.1016/j.neuron.2014.05.001 24853935

[29] Ceunen E Vlaeyen JWS Van Diest I. On the origin of interoception. Front Psychol. (2016) 7:743. 10.3389/fpsyg.2016.00743 27242642 PMC4876111

[30] D'Alessandro G Cerritelli F Cortelli P. Sensitization and interoception as key neurological concepts in osteopathy and other manual medicines. Front Neurosci. (2016) 10:100. 10.3389/fnins.2016.00100 27013961 PMC4785148

[31] Fairhurst MT Löken L Grossmann T. Physiological and behavioral responses reveal 9-month-old infants' sensitivity to pleasant touch. Psychol Sci. (2014) 25:1124–31. 10.1177/0956797614527114 24681587 PMC4017181

[32] Cerritelli F Chiacchiaretta P Gambi F Ferretti A. Effect of continuous touch on brain functional connectivity is modified by the operator's tactile attention. Front Hum Neurosci. (2017) 11:368. 10.3389/fnhum.2017.00368 28775685 PMC5517483

[33] Edwards DJ Young H Johnston R. The immediate effect of therapeutic touch and deep touch pressure on range of motion, interoceptive accuracy and heart rate variability: a randomized controlled trial with moderation analysis. Front Integr Neurosci. (2018) 12:41. 10.3389/fnint.2018.00041 30297988 PMC6160827

[34] Woo SH Lumpkin EA Patapoutian A. Merkel cells and neurons keep in touch. Trends Cell Biol. (2015) 25:74–81. 10.1016/j.tcb.2014.10.003 25480024 PMC4312710

[35] Dong YL Chauhan M Green KE Vegiraju S Wang HQ Hankins GD. Circulating calcitonin gene-related peptide and its placental origins in normotensive and preeclamptic pregnancies. Am J Obstet Gynecol. (2006) 195:1657–67. 10.1016/j.ajog.2006.04.006 16996466

[36] Mustafa L Islami P Shabani N Jashanica A Islami H. Response of smooth bronchial musculature in bronchoconstrictor substances in newborn with lung atelectasis at the respiratory distress syndrome (RDS). Med Arch. (2014) 68:6–9. 10.5455/medarh.2014.68.6-9 24783902 PMC4272476

[37] Ng QX Venkatanarayanan N Ho CYX Sim WS Lim DY Yeo WS. Selective serotonin reuptake inhibitors and persistent pulmonary hypertension of the newborn: an update meta-analysis. J Womens Health. (2019) 28:331–8. 10.1089/jwh.2018.7319 30407100

[38] Maksimovic S Baba Y Lumpkin EA. Neurotransmitters and synaptic components in the Merkel cell-neurite complex, a gentle-touch receptor. Ann NY Acad Sci. (2013) 1279:13–21. 10.1111/nyas.12057 23530998 PMC3638015

[39] Mulkey SB du Plessis AJ. Autonomic nervous system development and its impact on neuropsychiatric outcome. Pediatr Res. (2019) 85:120–6. 10.1038/s41390-018-0155-0 30166644 PMC6353676

[40] Mól N Kwinta P. Assessment of body composition using bioelectrical impedance analysis in preterm neonates receiving intensive care. Dev Period Med. (2015) 19:297–304. 26958693

[41] Kluckow M. The pathophysiology of low systemic blood flow in the preterm infant. Front Pediatr. (2018) 6:29. 10.3389/fped.2018.00029 29503814 PMC5820306

[42] Vrancken SL van Heijst AF de Boode WP. Neonatal hemodynamics: from developmental physiology to comprehensive monitoring. Front Pediatr. (2018) 6:87. 10.3389/fped.2018.00087 29675404 PMC5895966

[43] Patural H Pichot V Flori S Giraud A Franco P Pladys P. Autonomic maturation from birth to 2 years: normative values. Heliyon. (2019) 5:e01300. 10.1016/j.heliyon.2019.e01300 30899829 PMC6407160

[44] DiPietro JA Hodgson DM Costigan KA Hilton SC Johnson TR. Fetal neurobehavioral development. Child Dev. (1996) 67:2553–67. 10.2307/1131640 9022256

[45] Schmidt A Schneider U Witte OW Schleußner E Hoyer D. Developing fetal motor-cardiovascular coordination analyzed from multi-channel magnetocardiography. Physiol Meas. (2014) 35:1943–59. 10.1088/0967-3334/35/10/1943 25229562

[46] Esteves JE Spence C Diagnostic palpation and decision making: a neurocognitive model of expertise. Russian Osteopathic J. (2014) 1–2:92–109.

[47] Clark A. Whatever next? Predictive brains, situated agents, and the future of cognitive science. Behav Brain Sci. (2013) 36:181–204. 10.1017/S0140525X12000477 23663408

[48] Friston K. The free-energy principle: a unified brain theory?. Nat Rev Neurosci. (2010) 11:127–38. 10.1038/nrn2787 20068583

[49] Lederman SJ Klatzky RL. Haptic perception: a tutorial. Atten Percept Psychophys. (2009) 71:1439–59. 10.3758/APP.71.7.1439 19801605

[50] AACOM Glossary of Osteopathic Terminology. American Association of Colleges of Osteopathic Medicine. (2011) Available online at: https://www.aacom.org/docs/default-source/insideome/got2011ed.pdf (accessed March 23, 2020).

[51] Skedung L Arvidsson M Chung JY Stafford CM Berglund B Rutland MW. Feeling small: exploring the tactile perception limits. Sci Rep. (2013) 3:2617. 10.1038/srep02617 24030568 PMC3771396

[52] Zhang Z Li C Zhang J Huang Q Go R Yan T. Discrimination threshold for haptic volume perception of fingers and phalanges. Atten Percept Psycho. (2018) 80:576–85. 10.3758/s13414-017-1453-z 29218598

[53] Bergmann Tiest WM Kahrimanovic M Niemantsverdriet I Bogale K Kappers AM. Salient material properties and haptic volume perception: the influences of surface texture, thermal conductivity, and compliance. Atten Percept Psycho. (2012) 74:1810–8. 10.3758/s13414-012-0372-2 22972632

[54] Dhong C Miller R Root NB Gupta S Kayser LV Carpenter CW. Role of indentation depth and contact area on human perception of softness for haptic interfaces. Sci Adv. (2019) 5:eaaw8845. 10.1126/sciadv.aaw8845 31497646 PMC6716960

[55] Kappers AML Bergmann Tiest WM Haptic perception. WIREs Cogn Sci. (2013) 4:357–74. 10.1002/wcs.1238 26304224

[56] Friedman RM Hester KD Green BG LaMotte RH. Magnitude estimation of softness. Exp Brain Res. (2008) 191:133–42. 10.1007/s00221-008-1507-5 18679665 PMC2574806

[57] Guimberteau JC Armstrong C Architecture of Human Living Fascia. The Extracellular Matrix and Cells Revealed through Endoscopy. Edinburgh: Handspring Publishing (2015). p. 232.

[58] Barker PJ Briggs CA Bogeski G. Tensile transmission across the lumbar fasciae in unembalmed cadavers: effects of tension to various muscular attachments. Spine. (2004) 29:129–38. 10.1097/01.BRS.0000107005.62513.32 14722403

[59] Vleeming A Pool-Goudzwaard AL Stoeckart R van Wingerden JP Snijders CJ. The posterior layer of the thoracolumbar fascia: its function in load transfer from spine to legs. Spine. (1995) 20:753–8. 10.1097/00007632-199504000-00001 7701385

[60] Chaudhry H Bukiet B Ji Z Stecco A Findley TW. Deformations experienced in the human skin, adipose tissue, and Fascia in osteopathic manipulative medicine. J Am Osteopath Assoc. (2014) 114:780–7. 10.7556/jaoa.2014.152 25288713

[61] Metzger A Drewing K. Memory influences haptic perception of softness. Sci Rep. (2019) 9:14383. 10.1038/s41598-019-50835-4 31591427 PMC6779751

[62] Dupin L Hayward V Wexler M. Generalized movement representation in haptic perception. J Exp Psychol Hum Percept Perform. (2017) 43:581–95. 10.1037/xhp0000327 28080111

[63] Bannigan K Watson R. Reliability and validity in a nutshell. J Clin Nurs. (2009) 18:3237–43. 10.1111/j.1365-2702.2009.02939.x 19930083

[64] Cerritelli F Martelli M Renzetti C Pizzolorusso G Cozzolino V Barlafante G. Introducing an osteopathic approach into neonatology ward: the NE-O model. Chiropr Man Ther. (2014) 22:18. 10.1186/2045-709X-22-18 24904746 PMC4046173

